# Supplementary material for: Hepatitis C Virus Improves Human Tregs Suppressive Function and Promotes Their Recruitment to the Liver
Source: Cells. 2019 Oct 22;8(10):1296. doi: 10.3390/cells8101296 (PMC6829901; doi:10.3390/cells8101296)
Supplement: Supplementary file 1 [file cells-08-01296-s001.pdf]

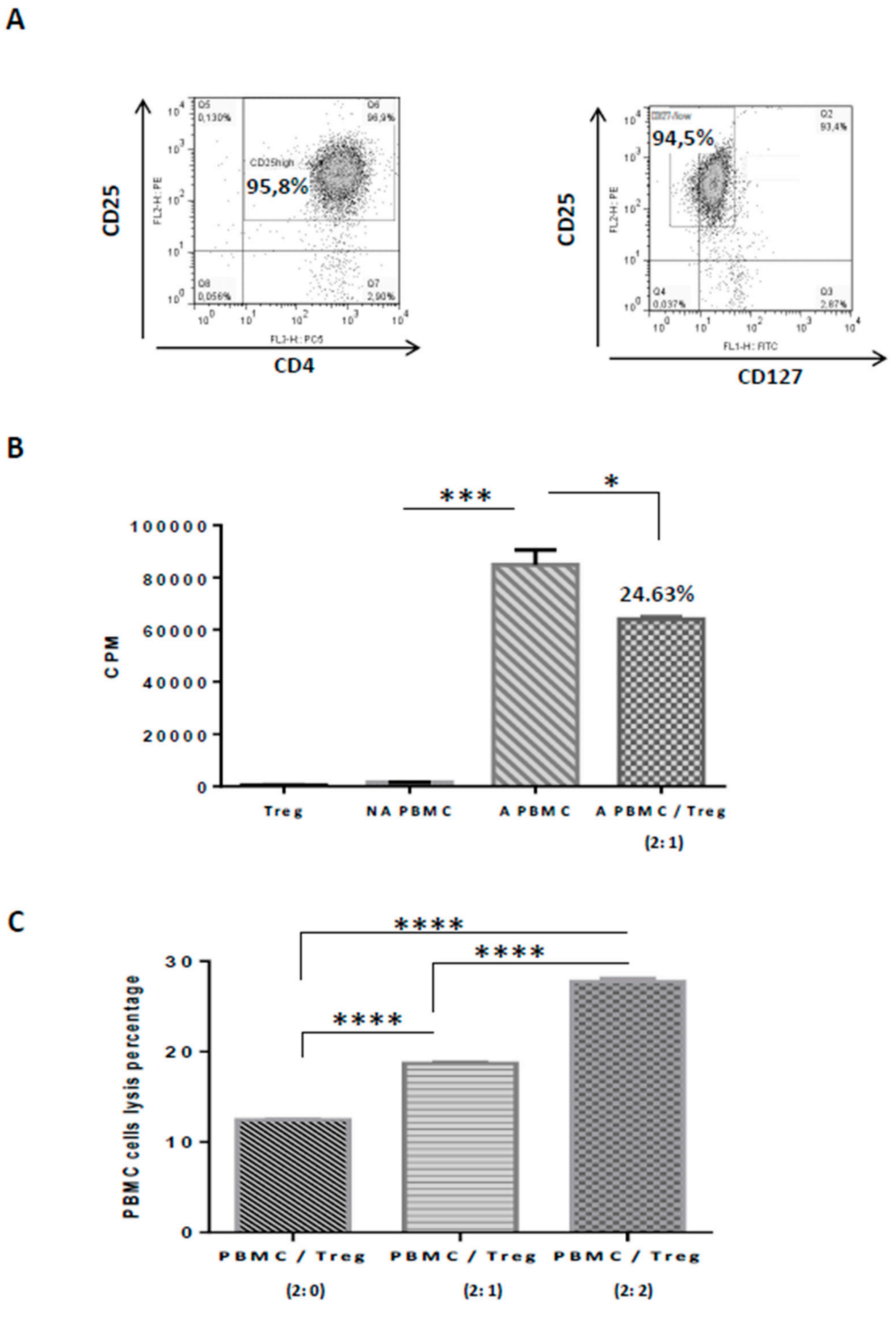

Figure S1. Phenotypic and functional characterization of freshly isolated natural regulatory T cells. Representative dot plot of triple stained CD4+CD25+/highCD127-/low Tregs after magnetic bead-isolation of 10 independent experiments (**A**). Based on CD4 and CD25 protein expression, the number in the upper-right quadrant indicates the percentage of CD4+CD25high Tregs which is superior to 95%. More than 94% of these freshly isolated Tregs are CD25highCD127-/low (upper-left quadrant). The suppressive activity of Tregs was addressed by using a model of lymphocyte reaction (MLR) by co-culture of autologous PBMC and Tregs at a 2:1 ratio in activated condition in the presence of plate-bound anti-human CD3 mAb (1µg/mL) and soluble mouse anti-human CD28 mAb (100ng/mL). Proliferation was

measured using [3H]-thymidin incorporation assays during the last 18 hours and values were obtained as counts per minute (cpm). Assays were performed after 48h and results revealed that isolated Tregs possess immunosuppressive capacity and significantly decrease of PBMC proliferation for around 24% (**B**). These representative results of 10 independent experiments are expressed as mean of cpm values of triplicate ± standard error of the mean (SEM) bars. The ability of Tregs to induce PBMC cells lysis was assessed by using a metabolic assay by co-culture of autologous PBMC and Tregs at 2:0, 2:1 and 2:2 ratios in activated condition (**C**). PBMC lysis percentage was measured by luminescence and normalized with the plate background value. Assays were performed after 48h of PBMC culture with Tregs. Tests were performed in duplicate in 3 independent experiments and results were expressed as percentage of PBMC cell lysis ± SEM bars.

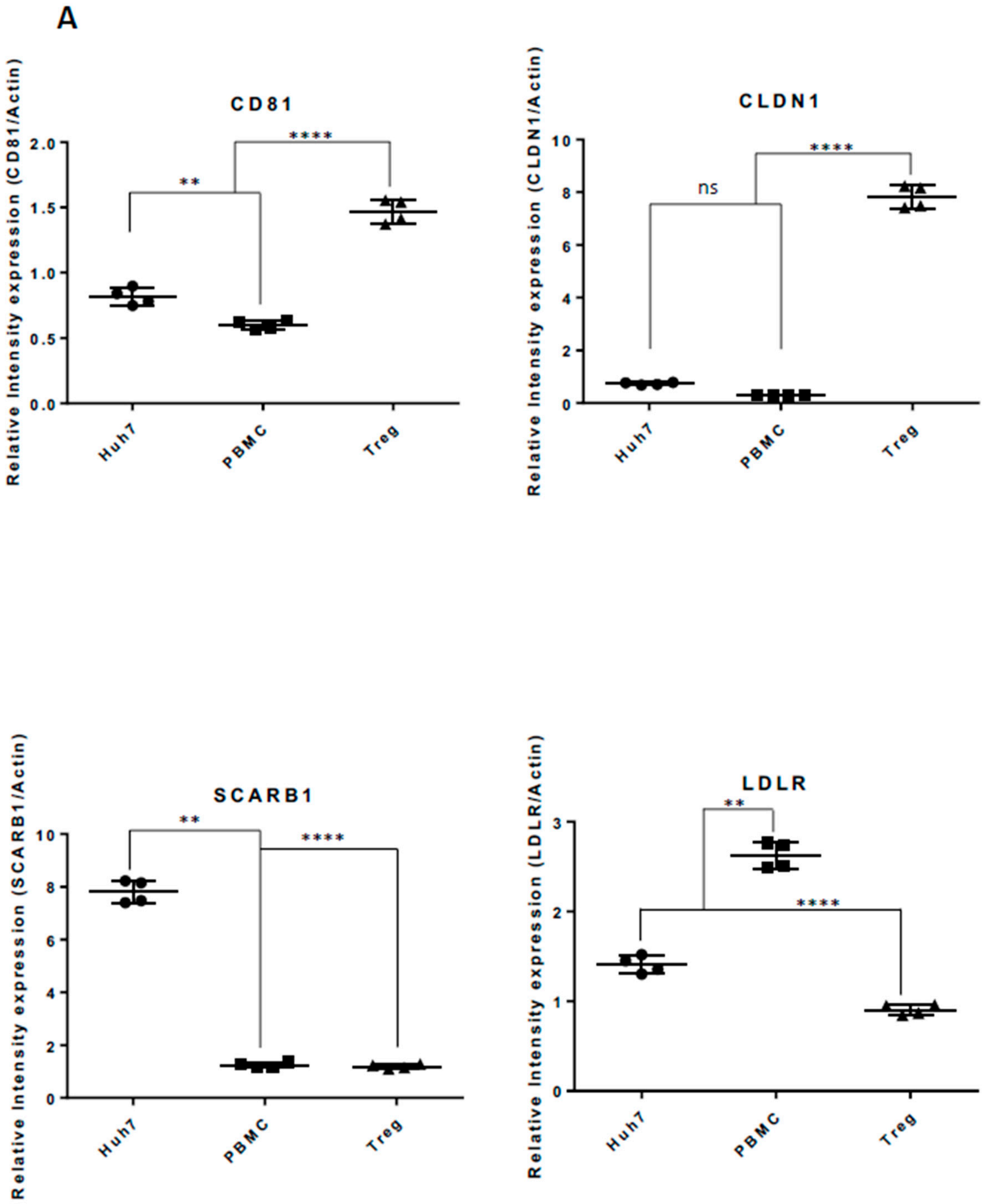

Figure S2. Tregs possess the classical HCV entry receptors: CD81, CLDN1 and LDLR. Relative total protein expression of HCV receptors detected by western-blot (**A**) and relative membranous protein expression of HCV receptors detected by FACS (**B**) on Huh7, PBMCs and Tregs. Results are the mean of four independent experiments ± SEM, statistical analysis represent the comparison in between Huh7 and either PBMCs or Tregs. ns = non-significant.

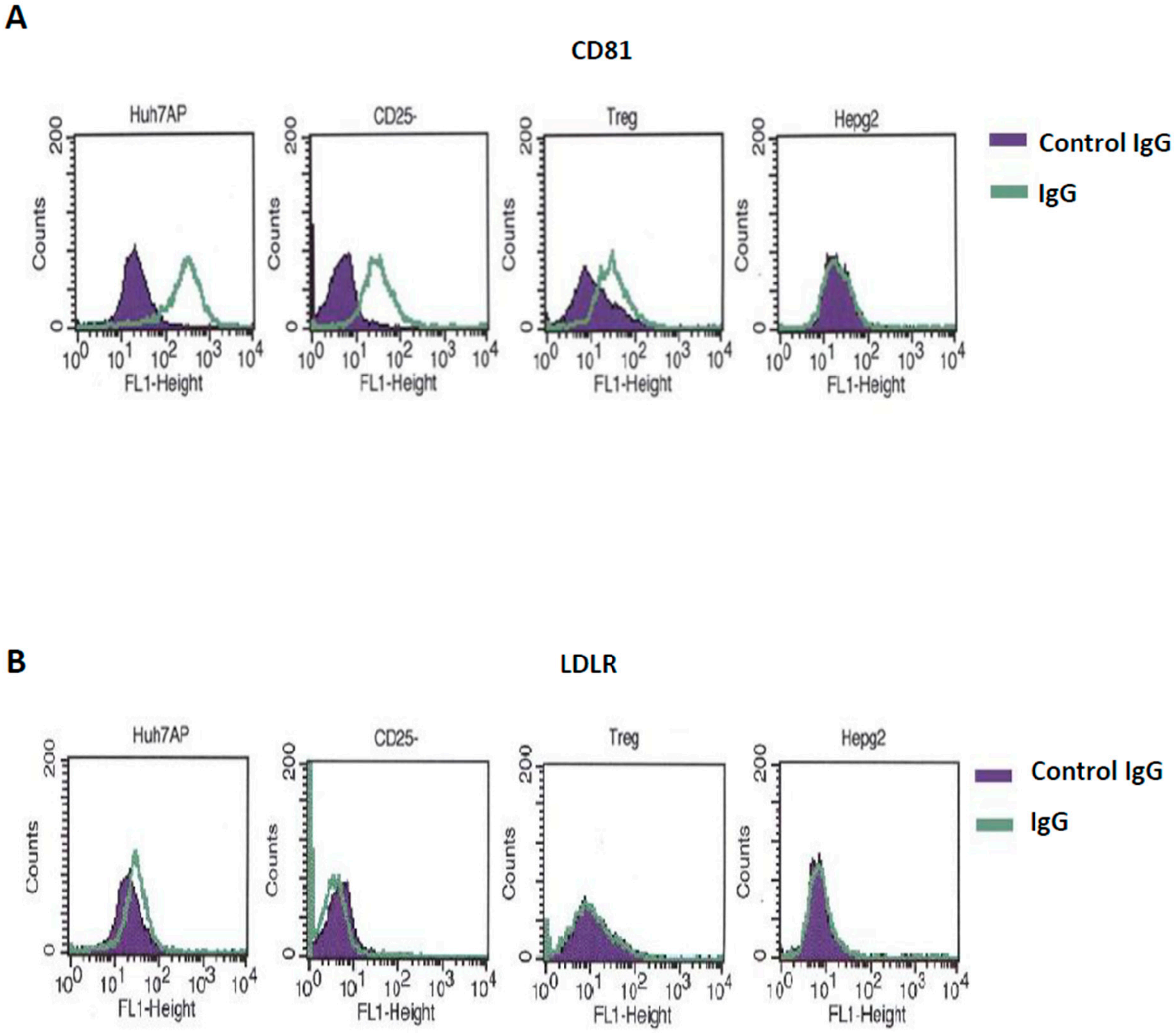

Figure S3. HepG2 cells do not express the HCV receptors CD81 and LDLR. Facs analyses were performed to evaluate the expression of HCV receptors CD81 (**A**) and LDLR (**B**) on Huh7 (HuhAP), Tconv (CD25-), Treg and HepG2 cells used as negative controls. Cells were labeled with either a specific antibody (IgG) or a relative isotype control (Control IgG). Results are representative of at least 5 independent experiments and are expressed in histograms displaying the percentage of cells positive for protein labeling compared to isotype control.

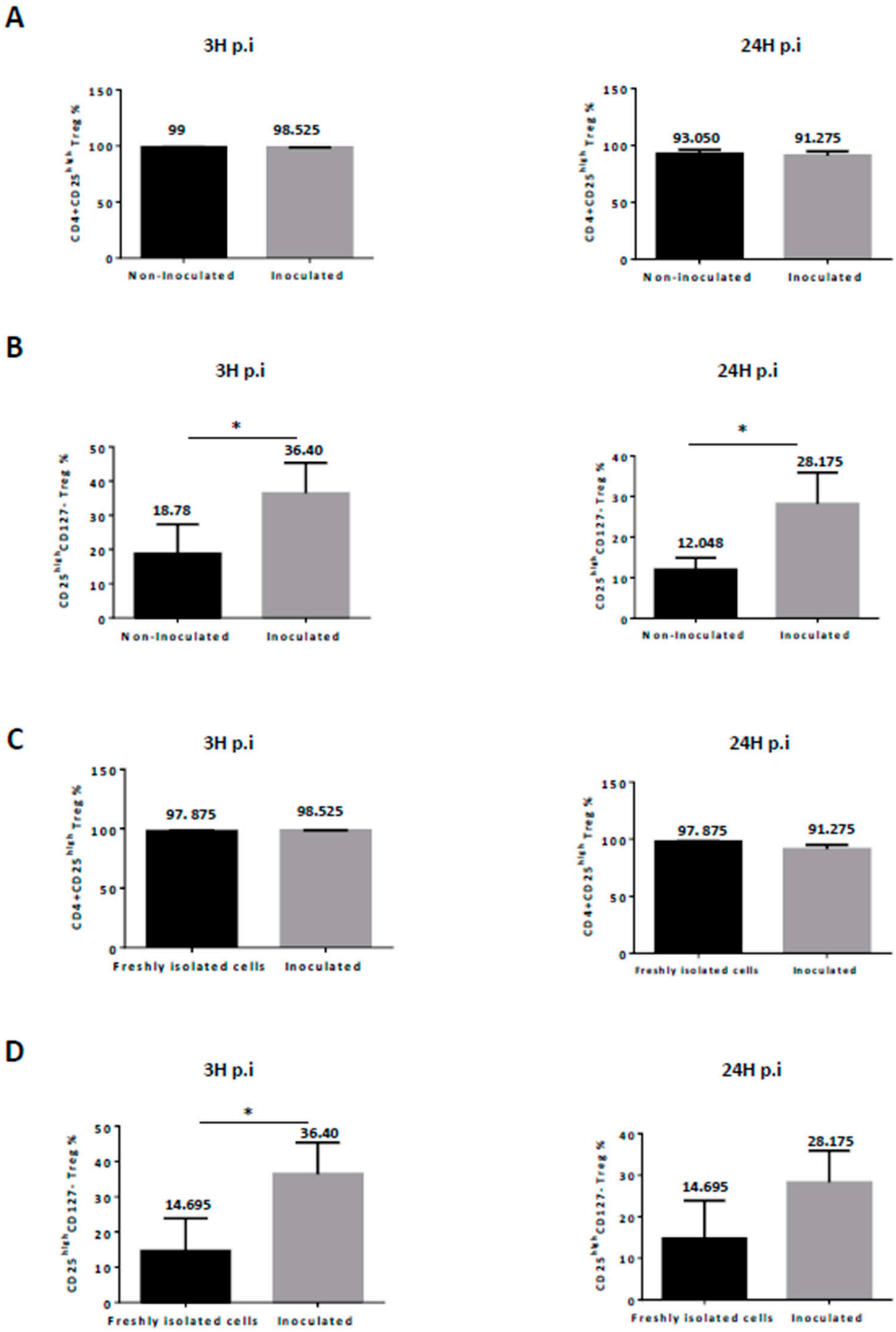

Figure S4. Impact of HCV inoculation on the frequency of suppressive Treg phenotype. FACS analyses were performed to evaluate the impact of HCV inoculation on the frequency of suppressive Tregs. CD4 + CD25high and CD25highCD127− T cells subsets were quantify between inoculated Tregs (light grey bars) vs non-inoculated Tregs (black bars) at both 3H p.i and 24H p.i (**A**,**B**). We also quantify these subsets between inoculated Tregs (light grey bars) versus freshly isolated cells (black bars) at both 3H p.i and 24H p.i (**C**,**D**). Results are expressed as means of percentage of the double stained population of 4 independent experiments ± SEM bars.

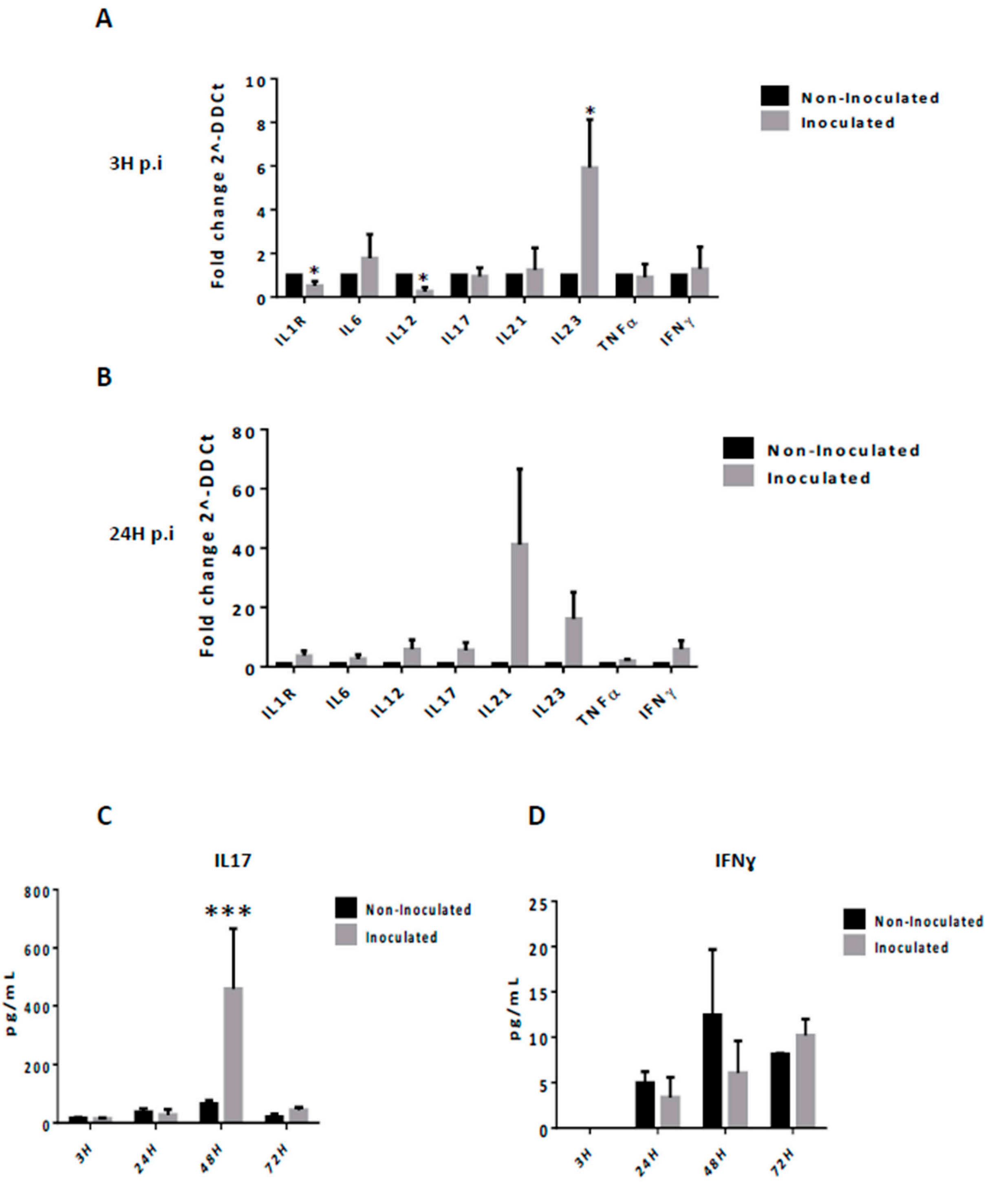

Figure S5. Impact of Tregs on the secretion of inflammatory cytokines afetr HCVcc inoculation. Tregs were handled in activated condition and were cultured in the presence or in the absence of HCV particles. Gene expression of inflammatory factors IL-1R, IL-6, IL-12, IL-16, IL-21, IL-23, TNFα and IFNɣ are expressed as means of 3 and 5 independent experiments (respectively 3H p.i and 24H p.i) (**A**,**B**). These gene expressions are normalized by using GADPH, β-actin, 18s and HPRT mRNA as housekeeping-gene before being reported to control and results are presented as fold change ($2^{-\Delta\Delta Ct}$) ± SEM bars comparing inoculated Tregs (light grey bars) versus non-inoculated Tregs (dark bars). Secretion of inflammatory cytokine IL-17 (**C**) and IFNɣ (D) were investigated by ELISA assays. Results are expressed as

mean of 3 independent experiment and presented in pg/mL ± SEM bars comparing secretion by inoculated Tregs (light grey bars) versus non-inoculated Tregs (dark bars).

Table 1. RT-Q-PCR Primers sequences.

**Genes**	**primer sequences**	
	**Forward**	**Reverse**
**CD81**	TGTATCTGGAGCTGGGAGACAAG	CCAGGAAGCCAACGAACATC
**SCARB1**	ATGGAACTTCTGGGCAAAG	CTTCAAACACCCCTGACTCC
**CLDN1**	GGTCAGGCTCTCTTCATCGG	GTTTTGGATAGGGCCTTGGT
**LDLR**	ACTGGTGTCAGAGGACCACC	CAAAGGAAGACGAGGAGCAC
**OCLN**	GGCCTCTTGAAAGTCCACCTC	CGAACATGCATCTCTCCACCA
**EGFR**	AGCTCTTCGGGGAGCAGCGA	ACTCGTGCCTTGGCAAACTTTCT
**CD5**	GAGCTCAATCATCTGCTACGGA	TTGTCGTTGGAGGTGTTGTCTT
**CD4**	GGGAAATCAGGGCTCCTTCTTA	TGGTCCCAAAGGCTTCTTCTT
**IL2RA (CD25)**	GGGACTGCTCACGTTCATCA	TTCAACATGGTTCCTTCCTTGTAG
**IL7R (CD127)**	GCAAGATACGTTTCCTCAGCAAC	TCCAAAGCTTTCTGGAGTGATGA
**FOXP3**	TCACCTACGCCACGGTCA	CACAAAGCACTTGTGCAG
**CTLA4**	TTCTTCTCTTCATCCCTGTCTTCT	GAGATGCATACTCACACACAAAGCT
**LAG3**	TGGCTTCAACGTCTCCATCA	CCCACCCTGGAACCTGCT
**IL2**	ACCAGGATGCTCACATTTAAGTTTTAC	TCCAGAGGTTTGAGTTCTTCTTCTAGA
**IL4**	CACAAGCAGCTGATCCGATTC	TTCCAAGAAGTTTTCCAACGTACTC
**PRDM1 (BLIMP1)**	GACGGGGGTACTTCTGTTCA	GGCATTCTTGGGAACTGTGT
**BCL6**	CTGCAGATGGAGCATGTTGT	CACCCGGGAGTATTTCTCAG
**IL15**	TTTCCATCCAGTGCTACTTGTGTT	CATTCACCCAGTTGGCTTCTGT
**IL10**	GAGAACCAAGACCCAGACATCAA	CCACGGCCTTGCTCTTGTT
**IL24**	AAGCCTTCTGGGCTGTGAAA	TGTGGACAAGGTAACAGCTCTCA
**IL12A (p35)**	CCTTCACCACTCCCAAAACCT	TGGTAAACAGGCCTCCACTGT
**EBI3**	CCCCGCCACTGCCACAATGA	GCCCTCCAACAGGTGTCCCG
**GZMB**	CGCCCCTACATGGCTTATCTT	CCCCCAAGGTGACATTTATGG
**TGFB1**	CGAGCCTGAGGCCGACTAC	CGGAGCTCTGATGTGTTGAAGA
**IL1R(1)**	CCACAAGGCCTGTGATTGTG	TCAACTGGCCGGTGACATTA
**IL6**	ATGTAGCCGCCCCACACA	CCAGTGCCTCTTTGCTGCTT
**IL12B (p40)**	CTTTCTAAGATGCGAGGCCAAG	AGAGGTGTAGCACTCCGCAC
**IL17(A)**	TCCTGGGAAGACCTCATTGG	AGAATTTGGGCATCCTGGATT
**IL21**	GATCGCCACATGATTAGAATGC	AGGAAAAAGCTGACCACTCACAGT
**IL23(A)**	GTGGGACACATGGATCTAAGAGAA	AAATCAGACCCTGGTGGATCCT
**IFNG**	ATGTAGCGGATAATGGAACTC	GACATTCAAGTCAGTTACC
**TNFA**	ATCTTCTCGAACCCCGAGTGA	GGAGCTGCCCCTCAGCTT
**CCL2**	GCTCATAGCAGCCACCTTCATT	ACTTGCTGCTGGTGATTCTTCTATA
**CCL3**	ATGGCTCTCTGCAACCAGTTCT	CGTCTCAAAGTAGTCAGCTATGAAATTC
**CCL 4**	GACTGTCCTGTCTCTCCTCATGCTA	AAGCTTCCTCGCGGTGTAAGA
**CCL7**	ATGGCTCTCTGCAACCAGTTGT	CGTCTCAAAGTAGTCAGCTATGA
**CCL17**	GGGCTTCTCTGCAGCACATC	GGTACCACGTCTTCAGCTTTCTAA
**CCL20**	GGGCTTCTCCTGGCTGCTTTG	GAATACGGTCTGTGTATCCAAGACA
**CCL22**	TTGCTGTGGCGCTTCAAG	CAGACGGTAACGGACGTAATCAC
**CXCL9**	GGCATCATCTTGCTGGTTCTG	GGTGGATAGTCCCTTGGTTGGT
**CXCL11**	TTGGCTGTGATATTGTGTGCTACA	TGCCACTTTCACTGCTTTTACC
**CXCL16**	ACACGAGGTTCCAGCTCCTTT	CAATCCCCGAGTAAGCATGTC
**CCR2**	GATCTGCTTTTTCTTATTACTCTCCCA	TCCGCCAAAATAACCGATGT
**CCR3**	GGTACCACATCCTACTATGATGACGT	CCACAGTGAACACCAGGGAGT
**CCR4**	CCACCCTCGATGAAAGCATATAC	TGCCTTGATGCCTTCTTTGG
**CCR5**	GTCAAGTCCAATCTATGACATCAATTATT	CGGGCTGCGATTTGCTT
**CCR6**	GTCAAGTCCAATCTATGACATCAATTATT	-CGGGCTGCGATTTGCTT
**CXCR3**	TCTTCCTATGACTATGGAGAAAACGA	CGGTCGAAGTTCAGGCTGAA
**CXCR4**	TCATGGGTTACCAGAAGAAACTGA	GAAGTTCCCAAAGTACCAGTTTGC

**CXCR6**	ACTATGGGTTCAGCAGTTTCAATG	CAGGTACATGCAGGGCAGAA
**ACTB**	CACGGCATCGTCACCAACT	GCCTGCTTCACCACCTTCTTGATGTC
**GAPDH**	GCCAAGGTCATCCATGACAACTTTGG	GCCTGCTTCACCACCTTCTTGATGTC
**HRPT**	CCCTGGCGTCGTGATTAG	ATGGCCTCCCATCTCCTT
**UFD1 (ubiquitin)**	CCGACCACAGTGGCTATGC	CCTCTTTTAATATCTCCAGGCTTGA
**RNA 18S**	TCAAGAACGAAAGTCGGAGG	GGACATCTAAGGGCATCACA

Table S2. Western Blot Density Analysis (A) and protein expression of HCV receptor in Huh7 to the expression within PBMC and Treg (B) (n = 4 donors).

		**A.** Ratio = Net loading x protein/Net loaded Actin.			
		**RUN 1***	**RUN 2**	**RUN 3**	**RUN 4**
**CD81**	Huh7	0,7810	0,8408	0,7498	0,8982
	PBMC	0,5748	0,6257	0,5690	0,6380
	Treg	1,4130	1,5402	1,3706	1,5543
**CLDN1**	Huh7	0,0318	0,0350	0,0309	0,0353
	PBMC	0,7146	0,7718	0,6860	0,7860
	Treg	0,2823	0,2992	0,2794	0,3246
**SCARB1**	Huh7	7,4816	8,1549	7,4068	8,2297
	PBMC	1,1819	1,3000	1,1465	1,3592
	Treg	1,1422	1,2222	1,0965	1,2678
**LDLR**	Huh7	1,3569	1,4519	1,3026	1,5198
	PBMC	2,5115	2,7375	2,4864	2,7627
	Treg	0,8690	0,9559	0,8430	0,9472

*data used as the representative one for figure1.

	**B. Statistical Analysis ONE WAY ANOVA, Tukey's multiple comparisons test**				
	**Mean Diff**	**95% CI of diff,**	**Significant Status**	**Summary**	**COMPARISON**
**CD81**	0,2156	0,1464 to 0,2848	**Yes**	**	*Huh7 vs. PBMC*
	−0,6521	−0,7248 to −0,5793	**Yes**	****	*Huh7 vs. Treg*
	-0,8677	−0,9870 to −0,7483	**Yes**	****	*PBMC vs. Treg*
**CLDN1**	0,4432	−0,05502 to 0,9415	**No**	**ns**	*Huh7 vs. PBMC*
	−7,079	−7,577 to −6,580	**Yes**	****	*Huh7 vs. Treg*
	−7,522	−8,020 to −7,024	**Yes**	****	*PBMC vs. Treg*
**SCARB1**	6,571	6,056 to 7,087	**Yes**	****	*Huh7 vs. PBMC*
	6,636	6,121 to 7,151	**Yes**	****	*Huh7 vs. Treg*
	0,06472	−0,4505 to 0,5799	**No**	**ns**	*PBMC vs. Treg*
**LDLR**	−1,217	−1,426 to −1,007	**Yes**	****	*Huh7 vs. PBMC*
	0,504	0,2945 to 0,7135	**Yes**	***	*Huh7 vs. Treg*
	1,721	1,511 to 1,930	**Yes**	****	*PBMC vs. Treg*

Diff: difference; CI: confident interval; ns: non-significant, * indicate $p \le 0.05$, ** indicate $p \le 0.01$***, indicate $p \le 0.001$, **** indicate $p \le 0.0001$.

Table S3. FACS Analysis of the expression of HCV entry receptors (**A**), statistical analysis of protein expression in Huh7 vs. expression within PBMC and Treg (**B**) (n = 4 donors).

		A. Percentage of Marked Cells within population			
		**RUN 1***	**RUN 2**	**RUN 3**	**RUN 4**
**CD81**	***Huh7***	57,60	63,36	55,30	66,24
	PBMC	28,00	29,96	27,72	31,08
	Treg	47,60	51,88	46,17	52,36
**CLDN1**	***Huh7***	54,20	59,62	52,57	60,16
	PBMC	65,50	70,74	64,85	72,05
	Treg	89,70	95,08	88,80	103,16
**SCARB1**	***Huh7***	1,50	1,64	1,49	1,65
	PBMC	4,20	4,62	4,07	4,83
	Treg	2,33	2,49	2,24	2,59
**LDLR**	***Huh7***	2,08	2,23	2,00	2,33
	PBMC	2,74	2,99	2,66	3,01
	Treg	2,16	2,38	2,14	2,35

*data used as the representative one for figure 1.

		B. Statistical Analysis ONE WAY ANOVA , Tukey's multiple comparisons test				
		Mean Diff,	*95% CI of diff,*	*Significant Status*	*Summary*	**COMPARISON**
**CD81**	***Huh7***	0,4432	−0,05502 to 0,9415	**No**	ns	*Huh7 vs. PBMC*
	PBMC	−7,079	−7,577 to −6,580	**Yes**	****	*Huh7 vs. Treg*
	Treg	−7,522	−8,020 to −7,024	**Yes**	****	*PBMC vs. Treg*
**CLDN1**	***Huh7***	−11,64	−21,27 to −2,020	**Yes**	*	*Huh7 vs. PBMC*
	PBMC	−37,55	−47,17 to −27,92	**Yes**	****	*Huh7 vs. Treg*
	Treg	−25,9	−35,53 to −16,28	**Yes**	****	*PBMC vs. Treg*
**SCARB1**	***Huh7***	−2,864	−3,316 to −2,411	**Yes**	****	*Huh7 vs. PBMC*
	PBMC	−0,844	−1,297 to −0,3915	**Yes**	**	*Huh7 vs. Treg*
	Treg	2,019	1,567 to 2,472	**Yes**	****	*PBMC vs. Treg*
**LDLR**	***Huh7***	−0,692	−0,9916 to -0,3916	**Yes**	***	*Huh7 vs. PBMC*
	PBMC	−0,099	−0,3992 to 0,2008	**No**	ns	*Huh7 vs. Treg*
	Treg	0,5924	0,2924 to 0,8924	**Yes**	***	*PBMC vs. Treg*

Diff: difference; CI: confident interval; ns: non-significant. * indicate $p \leq 0.05$, ** indicate $p \leq 0.01$***, indicate $p \leq 0.001$, **** indicate $p \leq 0.0001$.

Table S4. Analysis of the ratio comparing expression levels of Tregs either to PBMCs or Huh7 for each employed technique of detection.

	**Ratio Treg/PBMC**		**Ratio Treg/Huh7**		
	**FACS**	**WB**	**FACS**	**WB**	**RT-QPCR**
**CD81**	1,70	2,44	0,82	1,80	2,09
**CLDN1**	1,38	0,40	1,66	8,91	1,02
**SCARB1**	0,54	0,95	1,54	0,15	0,08
**LDLR**	0,79	0,34	1,05	0,64	3,19

Table 5. Ranking levels of expression in Huh7, PBMC and Treg. (1 is the strongest and 3 the lowest).

	**CD81**		**CLDN1**		**SCARB1**		**LDLR**	
	WB	***FACS***	***WB***	***FACS***	***WB***	***FACS***	***WB***	***FACS***
**Huh7**	2	1	3	3	1	3	2	3
**PBMC**	3	3	1	2	2	2	1	1
**Treg**	1	2	2	1	3	1	3	3

WB: werstern-blot, FACS: flow cytometry, Treg: regulatrory T cells, PBMC: peripheral blood mononuclear cells.
